# Talking about treatment benefits, harms, and what matters to patients in radiation oncology: an observational study

**DOI:** 10.1186/s12911-019-0800-5

**Published:** 2019-04-11

**Authors:** Laurie Pilote, Luc Côté, Selma Chipenda Dansokho, Émilie Brouillard, Anik M. C. Giguère, France Légaré, Roland Grad, Holly O. Witteman

**Affiliations:** 1 Division of Radiation Oncology, Department of Medicine, CHU de Québec, 11, Côte du Palais, Quebec City, QC G1R 0A2 Canada; 2 Department of Family and Emergency Medicine, Faculty of Medicine, Laval University, Pavillon Ferdinand-Vandry 2881, 1050 avenue de la Médecine, Quebec City, QC G1V 0A6 Canada; 3 Office of Education and Professional Development, Faculty of Medicine, Laval University, Pavillon Ferdinand-Vandry 2881, 1050 avenue de la Médecine, Quebec City, QC G1V 0A6 Canada; 4 Population Health and Optimal Health Practices Research, 10 rue de l’Espinay, Hôpital Saint François d’Assise D6, Quebec City, QC G1L 3L5 Canada; 5 Quebec Excellence Centre on Aging, Research Centre of the CHU de Quebec, St-Sacrement Hospital, Local L2-08, 1050 chemin Sainte-Foy, Quebec City, QC G1S 4L8 Canada; 6 Centre de recherche sur les soins et les services de première ligne de l’Université Laval (CERSSPL-UL) CERSSPL-UL, 880, rue Père-Marquette, 3e étage, Québec, QC G1S 2A4 Canada; 7 Department of Family Medicine, Faculty of Medicine, McGill University, 5858, chemin de la Côte-des-Neiges, Montreal, QC H3S 1Z1 Canada; 8 Herzl Family Practice Centre, Jewish General Hospital, 3755 Côte-Ste-Catherine Road, E-740, Montreal, QC H3T 1E2 Canada

**Keywords:** Shared decision making, Patient-clinician communication, Risk communication, Values clarification

## Abstract

**Background:**

Shared decision making is associated with improved patient outcomes in radiation oncology. Our study aimed to capture how shared decision-making practices–namely, communicating potential harms and benefits and discussing what matters to patients–occur in usual care.

**Methods:**

We invited a convenience sample of clinicians and patients in a radiation oncology clinic to participate in a mixed methods study. Prior to consultations, clinicians and patients completed self-administered questionnaires. We audio-recorded consultations and conducted qualitative content analysis. Patients completed a questionnaire immediately post-consultation about their recall and perceptions.

**Results:**

11 radiation oncologists, 4 residents, 14 nurses, and 40 patients (55% men; mean age 64, standard deviation or SD 9) participated. Patients had a variety of cancers; 30% had been referred for palliative radiotherapy. During consultations (mean length 45 min, SD 16), clinicians presented a median of 8 potential harms (interquartile range 6–11), using quantitative estimates 17% of the time. Patients recalled significantly fewer harms (median recall 2, interquartile range 0–3, t(38) = 9.3, *p* < .001). Better recall was associated with discussing potential harms with a nurse after seeing the physician (odds ratio 7.5, 95% confidence interval 1.3–67.0, *p* = .04.) Clinicians initiated 63% of discussions of harms and benefits while patients and families initiated 69% of discussions about values and preferences (Chi-squared(1) = 37.8, *p* < .001). 56% of patients reported their clinician asked what mattered to them.

**Conclusions:**

Radiation oncology clinics may wish to use interprofessional care and initiate more discussions about what matters to patients to heed Jain’s (2014) reminder that, “a patient isn’t a disease with a body attached but a life into which a disease has intruded.”

## Background

Health care has shifted from a paternalistic model to one of shared decision making, with patients encouraged to take an active role in decision making [1, 2]. Patients have unique knowledge of their own situation and preferences, and patient participation in decision making may improve their experience, health outcomes, and the quality of care they receive, as well as health care provider satisfaction and health system sustainability [3–8]. However, as in other specialties [9], work remains to be done to implement shared decision-making principles and practices in radiation oncology [7, 10–14].

The three core practices within shared decision making are identifying that a decision must be made, communicating the potential benefits and harms of options (including doing nothing yet), and incorporating what matters to the patient relevant to the decision [6, 15, 16].

This study aimed to observe real-world communication practices in radiation oncology in an urban academic medical center, their effects on patient recall of benefits and harms, and whether and how patients’ values and preferences entered the conversation.

## Methods

### Study design and context

During a 4-week period from November to December 2013, we invited clinicians and patients at a single academic medical center to participate in this observational study. The study site, the Hôtel-Dieu de Quebec, is part of an academic medical center affiliated with Laval University in Quebec City, Canada, the “Centre hospitalier universitaire (CHU) de Québec-Université Laval,” and provides radiation oncology care to over 4400 patients each year from a large geographic area of the province of Quebec. Approval to conduct the study was obtained from the CHU de Québec-Université Laval’s Institutional Review Board.

### Participant eligibility

All clinicians seeing patients during the study recruitment period were eligible to participate in the study. Only patients presenting for an intake radiation oncology appointment with a participating clinician during the study recruitment period were eligible for the study. Participation proceeded if all parties who would be present in the consultation room, including clinicians, patients, and any accompanying family members agreed to participate in the study. Patients were excluded if they and/or their accompanying family member declined to participate, or if they were unable to complete a brief questionnaire in French or English.

### Participant recruitment

The lead author (LP), a medical resident in the clinic, first recruited clinicians and then invited a convenience sample of patients seeing participating clinicians to join the study upon their arrival for scheduled appointments. We aimed for 40 patients in total, deeming this sample size feasible within project scope and sufficient to enable qualitative and quantitative exploratory analyses.

### Data collection

Participating clinicians each completed a brief questionnaire about their decision-making style preferences and socio-demographic characteristics. Participating patients first completed a written questionnaire about their socio-demographic characteristics, and original English or translated French versions of validated measures of health literacy [17] and subjective numeracy [18]. Each clinical consultation was unobtrusively audio-recorded by the lead researcher (LP) who briefly entered the room to turn the recorder on immediately before the consultation and off after the patient left the room. No member of the research team was otherwise present during medical consultations. After the visit, patients completed a second written questionnaire with a validated measure of their decision-making style preferences [19], questions regarding what they recalled of the potential benefits and harms discussed by clinicians, whether or not their clinicians had asked them about what mattered to them, and an original English or translated French version of a validated patient-reported measure of shared decision making (the 9-item Shared Decision Making Questionnaire, SDM-Q-9) [20]. We asked patients about their decision-making style preferences only after the clinical consultation to reduce the possibility that the question might influence their behavior during the consultation.

### Data analysis: recordings of clinical encounters

All audio recordings were transcribed verbatim by the lead author (LP). We conducted a thematic analysis of transcripts in NVivo 10 (QSR International, Cambridge, MA) using inductive and deductive approaches and Braun and Clarke’s method of thematic analysis [21].

To address our aim of describing how clinicians communicate about potential benefits and harms, we used thematic categories from an established taxonomy of levels of risk communication [22] and an inventory of best practices in risk communication [23]. First, we analyzed whether or not clinicians used numerical estimates to discuss benefits and harms, classifying statements within one of three approaches: words alone; numbers alone; or a combination of words and numbers. Second, because there is always uncertainty about whether or not a treatment will have the desired effect or cause a side effect, we analyzed whether or not clinicians communicated any forms of uncertainty [24] within potential benefits and harms. We also created lists of all benefits and harms mentioned by clinicians, patients and family members in each clinical visit. Data from the transcripts about harms and benefits were first extracted by a resident in radiation oncology (LP) and independently verified by a second resident in radiation oncology (EB) to ensure medical accuracy in coding.

To address our aim of describing how values and preferences are discussed, we drew on the medical decision making literature and defined values as attributes that matter to patients or their family members, and preferences as inclinations toward or away from a medical option [25]. We developed a thematic structure around values and preferences iteratively via repeated examination and discussion of the data and thematic structure by 4 researchers on the team (LP, LC, SCD, HW), two with expertise in qualitative research (LC, SCD) and two with content expertise (LP, HW), until we reached saturation of themes. All transcripts were then thematically analyzed by the lead author (LP) and verified by the senior author (HW), with 8 randomly-selected analyzed transcripts independently verified by the other 2 researchers (LC, SCD) for additional rigor. Any disagreements were discussed within the group of 4 and resolved by consensus.

We report frequencies of themes regarding what was discussed during the consultation, how clinicians described potential benefits and harms, and who initiated discussions within different themes. We compared who initiated discussions of different issues using Chi-squared tests.

### Data analysis: questionnaires

All questionnaire data were entered into a spreadsheet by an administrative assistant and verified by the lead author (LP). We combined these data with the lists of benefits and harms from the transcripts to create a dataset detailing benefits and harms mentioned by health care professionals and recalled by patients and families, either verbally during the consultation or written on post-visit questionnaires. A resident in radiation oncology (LP) verified all data to ensure that benefits and harms that were differently worded (e.g., clinician: “nausea”, patient: “sick to my stomach”) were appropriately matched; a second resident (EB) validated the verification.

### Data triangulation and analysis: recall of benefits and harms

To assess overall patient recall of benefits and harms, we considered benefits and harms either written on the post-visit questionnaires, or mentioned by patients or family members when asked by a nurse. We calculated descriptive statistics of how many benefits and harms health care professionals communicated to patients and family members, and how many the patients and family members recalled. We then conducted a paired t-test to compare the number of benefits and harms communicated to each patient with the number actually recalled.

In our qualitative analyses, we noted that sometimes when patients met with a nurse following their consultation with a physician, the nurse asked the patient what he or she recalled the physician saying. To explore whether this practice influenced patient recall as assessed by what they wrote on the post-visit questionnaire, we conducted a logistic regression of patients’ written recall of benefits and harms. We tested the predictor of whether the nurse had asked the patient what they recalled, controlling for age, sex, health literacy and numeracy.

### Data triangulation and analysis: discussions of values

We counted the number of independent statements initiated about values, and compared who (i.e. clinicians or patients and family) initiated statements of potential benefits and harms versus who initiated statements of values via a Chi-squared test.

We conducted all statistical analyses in R, version 3.2.1 (R Foundation for Statistical Computing, Vienna, Austria.)

## Results

### Study participants

Twenty-nine of the 30 clinicians who were invited (97%) agreed to participate: 11 radiation oncologists, 4 residents, and 14 nurses. Of 46 patients invited, 40 (87%) agreed to participate: 22 men (55%) and 18 women (45%) with a mean age of 64 years (standard deviation, SD 9, range 41–78). One participating patient completed the first questionnaire but was too tired to complete the second questionnaire; this patient’s socio-demographic data were included in the description of study participants but this person was removed from analyses of post-visit outcomes. There was wide variability among patients and clinicians regarding preferred decision-making style, although most clinicians and patients preferred either patient-led or shared decision making. See Table 1 for clinician and patient characteristics.

**Table 1:** Participant characteristics

		Oncologists (*n* = 11)	Nurses (*n* = 14)	Residents (*n* = 4)	Patients (*n* = 40)
Age	Mean (SD)	42 (9)	44 (13)	28 (2)	64 (9)
Sex	Male: N (%)	6 (55%)	0 (0%)	1 (25%)	22 (55%)
	Female: N (%)	5 (45%)	14 (100%)	3 (75%)	18 (45%)
Visible minority (Note 1)	N (%)	0 (%)	0 (%)	0 (%)	2 (5%)
Has a disability	N (%)	0 (%)	0 (%)	0 (%)	5 (13%)
Years in practice	Mean (SD)	10 (8)	22 (14)	5 (1)	–
Preferred decision-making style	Clinician-led: N (%)	2 (18%)	0 (0%)	0 (0%)	8 (21%)
	Shared: N (%)	4 (36%)	0 (0%)	2 (50%)	8 (21%)
	Patient-led: N (%)	5 (45%)	13 (93%)	2 (50%)	22 (56%)
	Did not answer: N (%)	0 (0%)	1 (7%)	0 (0%)	2 (5%)
Numeracy (scale 8–48)	Median (IQR)	40 (8)	37 (6)	42 (2)	35 (14)
Literacy (scale 3–18)	Median (IQR)	–	–	–	13 (4)
Education	No postsecondary degree: N (%)	–	–	–	22 (55%)
	Postsecondary degree: N (%)	–	–	–	17 (42.5%)
	Did not answer: N (%)	–	–	–	1 (2.5%)
Type of Cancer	Breast: N (%)	–	–	–	6 (15%)
	Prostate: N (%)	–	–	–	6 (15%)
	Lung: N (%)	–	–	–	5 (13%)
	Stomach, Rectal or Oesophagus: N (%)	–	–	–	4 (10%)
	Lymphoma: N (%)	–	–	–	4 (10%)
	Central Nervous System (CNS): N (%)	–	–	–	2 (5%)
	Head and Neck: N (%)	–	–	–	2 (5%)
	Melanoma: N (%)	–	–	–	1 (3%)
	Palliative care (various cancer types): N (%)	–	–	–	10 (25%)

(Note 1) Visible minority is a common Canadian term for self-reporting as a person of color. Quebec City’s population is very homogeneous, with most people being white francophones. *SD* Standard Deviation, *IQR* Interquartile Range

### Structure and nature of the discussions

Clinical consultations all followed a similar pattern. Patients first met with a nurse who reviewed their medical history and administered a standard clinic questionnaire to assess their symptoms and needs. Some patients (11/39, 28%) then met with a resident who discussed test or lab results and treatment options. After meeting with the nurse and possibly the resident, all patients met with a physician who established a care plan with the patient and their family members. Finally, the nurse returned to answer any remaining questions and review treatment procedures. In 19 of the 39 recorded consultations (49%), the nurse asked the patient what she or he recalled of the treatment side effects mentioned by the physician. The mean total duration of consultations was 45 min (SD 16 min).

The discussions during the consultations always included three main topics: a description of radiation therapy, a description of the potential benefits (or goals) of treatment, and the potential harms (or side effects) of treatment. In our thematic structure, statements could be assigned to only one of these themes. Across all such statements, 45% of discussions were devoted to a description of radiation therapy, 29% to its potential benefits, and 27% to its potential harms. When discussing potential harms, clinicians mentioned a median of 8 potential side effects (interquartile range, IQR 6–11) during each consultation. Residents mentioned the greatest number of side effects (median 9, IQR 6–12), followed by physicians (median 7, IQR 4–9) and then nurses (median 3, IQR 2–4). Clinicians initiated the majority of discussions in all three categories.

### Communicating potential benefits and harms

When discussing potential benefits, clinicians used words alone, numbers alone, and both numbers and words together in 83, 8 and 9% of statements, respectively. When discussing potential harms, clinicians used words alone, numbers alone, and both numbers and words together in 83, 1 and 16% of statements, respectively. Clinicians did not use numbers even when patients specifically asked for more information about probability. Clinicians attempted to convey the inherent uncertainty of the potential benefits and harms of treatment in 73% of statements about potential benefits and in 84% of statements about potential harms. Some used metaphors to illustrate ambiguity; for example, one physician compared forgoing adjuvant treatment to crossing a major, 6-lane local street with one’s eyes closed: “You might cross without incident or you could get hit.” (Clinician 3, Patient 44).

### Discussing patients’ and families’ values

To examine discussions of patients’ and families’ values and preferences relevant to radiation therapy, we identified statements about issues that could have an impact on a patient’s life or experiences during or after radiation therapy. Although we conducted this analysis inductively, an existing framework resonated in the data. This framework is taught in our academic medical center as four categories of what matters to patients and should be discussed in clinical consultations: *perceptions* (perceptions), *inquiétudes* (worries), *craintes* (fears) and *attentes* (expectations). The patient values that were discussed most frequently were: fears about radiation therapy, worries and fears about side effects, and expectations related to the potential benefits of radiation therapy. Fears about radiation therapy included issues related to quality of life and the experience of receiving radiation therapy, including important logistical issues such as how to arrange and pay for transportation and parking for frequent visits.

Comparing who initiated statements, we observed that clinicians initiated 63% of discussions about potential benefits and harms but only 31% of discussions about values. Patients and families initiated the remaining discussions, yielding a significant difference in who drives which aspects of the discussions (Chi-squared (1) = 37.8, *p* < .001).

### Patient recall

Results of the post-visit questionnaire show that most patients (31/39, 79%) responded that a decision had been made during the encounter. A minority of patients indicated that no decision had been made (4/39, 10%) or did not answer this question (4/39, 10%). Some patients wrote in the open comments section that there was no decision to be made because there were, “no other options.” (P32, P39, P40).

Patients recalled significantly fewer potential harms (side effects) than clinicians communicated. While clinicians had presented a median of 8 side effects (IQR 6–11), patients had a median total recall of 2 (IQR 0–3), t(38) = 9.3, *p* < .001.

Exploring patient recall in the post-visit questionnaires, two patients reported benefits (e.g., “being cured”) and nine patients (23%) reported potential harms. As a function of patient age, sex, health literacy, numeracy, and whether or not the nurse asked the patient what they recalled from their discussion with the physician and resident, we observed that only the nurse’s questioning was associated with patients recalling any harms on the questionnaire (odds ratio, OR 7.5, 95% confidence interval, CI: 1.3–67.0, *p* = .04.)

When asked whether the clinician had asked what mattered to them, 22/39 patients (56%) responded yes, 11/39 responded no (28%) and 5/39 were unsure (13%). Patient reports of the extent to which they experienced shared decision making (SDM-Q-9) were skewed left, with 11/39 patients (28%) reporting a perfect score of 100 on the 0–100 scale, a median score of 89, interquartile range 30, and scores ranging from 16 to 100.

## Discussion

This study aimed to describe how clinicians, patients and family members discuss the potential harms and benefits of radiation therapy and what matters to patients, and to observe the potential effects of such communication practices. Our study results lead to five main observations.

First, patients in our study did not recall nearly as much about potential benefits and harms as clinicians communicated. This is in keeping with previous research demonstrating that cancer patients’ recall of what their health care providers tell them during consultations can be limited [26]. Our study suggested that having nurses ask patients what the doctor and resident had said about the side effects of treatment was associated with better patient recall on questionnaires, independent of patient age, health literacy and numeracy. This result aligns with other research showing that such methods of “teach-back” may improve patient understanding and recall [27], including in patients with low health literacy [28]. However, we note that, as we would expect for a study of *n* = 39, this observed effect has a wide confidence interval and should therefore be interpreted with caution as a promising result that merits further study.

Second, we noted that clinicians in our study typically attempted to convey the inherent uncertainty around the potential benefits and harms of radiation therapy to patients and families. Most statements about benefits and harms included some suggestion of uncertainty. However, due to the low level of patient recall, it is difficult to ascertain whether or not such uncertainty is understood by patients. Clinicians presented uncertainty primarily using words, which could reflect a legitimate concern that patients may not understand probabilities, or an inability to cite an exact probability to patients with different types of cancer and different moderating clinical characteristics. Presenting uncertainty using only words is problematic because verbal labels such as “high risk” or “rare” are interpreted differently by different people [29, 30].

Third, our study suggests that health professionals make efforts to attend to what matters to patients but could do more to integrate patient values into health decisions. Slightly more than half of patients reported that their clinicians asked what mattered to them. Many statements about patients’ values were initiated by patients themselves or by family members. This is problematic because not all patients are comfortable bringing up such issues [31] and, when clinicians rather than patients broach issues relevant to a patient’s life, they are more likely to be addressed [32]. Discussing patients’ values and preferences may be particularly important in palliative care [33].

Fourth, devoting nearly half the time in consultations to providing standard information on radiation therapy raises the question of whether there might be other, more efficient ways to convey such information, freeing up clinicians’ time to focus on patient-specific details around potential benefits and harms, along with what matters to each individual patient and family.

Finally, as previously reported [34], in addition to clinical concerns, the logistics of treatment such as transportation, appointment frequency, and parking matter to radiation oncology patients. Attending to such aspects of the patient experience is part of providing patient-centered care.

Our methods and findings are consistent with those from a study in the Netherlands conducted by Kunneman and colleagues focusing specifically on preoperative radiotherapy for rectal cancer [35, 36]. The authors found that radiation oncologists mentioned a median of 7 benefits or side effects (range 2–12) of preoperative radiotherapy in each consultation. Unlike our study, radiation oncologists used numbers 59% of the time when describing probabilities, versus 17% in our study. In their study, clinicians initiated discussions of values and preferences in 22% of consultations, which is comparable to our assessment that clinicians initiated 31% of discussions of what matters to patients. Both studies were undertaken in academic medical centers, suggesting that differences in numeric risk communication may reflect cultural differences between the Netherlands and Quebec, Canada or may reflect Kunneman et al.’s focus on a single type of cancer. As in our study, post-visit questionnaires revealed that patients demonstrated limited understanding of the potential side effects of radiation treatment.

Our study had several strengths and limitations. We had high study participation rates by both clinicians and patients, which may demonstrate a shared interest in improving patient-clinician communication. Our data collection methods provided us with rich data to explore how communication occurs in routine clinical radiation oncology care, and the effects of such communication patterns for patients. Our single study site, however, limits generalizability of our results, and our decision to use both qualitative and quantitative methods meant that, because of the time required for qualitative analyses, we could not recruit a large enough sample for well-powered quantitative analyses. Additionally, in assessing patient recall immediately after the clinic visit, we aimed to maximize data completeness and response rate. However, this choice may have compromised our ability to fully capture patient recall because patients might have been too tired or overwhelmed to write down everything they recalled. It is also possible that patients may have recalled even less had we assessed recall several weeks after their appointment.

## Conclusions

In conclusion, our study suggests that to improve patient knowledge about potential benefits and harms in radiation oncology, a promising approach may be to work within a model of interprofessional care and have a nurse or other professional discuss with patients what they were told. To better integrate patient values into clinical discussions, clinicians who do not already do so may wish to explicitly ask patients and families about their perceptions, worries, fears, and expectations. Ensuring that these elements are covered within consultations may help address the reality that, “a patient isn’t a disease with a body attached but a life into which a disease has intruded” [37].

## Abbreviations

CHU: Centre hospitalier universitaire
CI: Confidence interval.
IQR: Interquartile range
OR: Odds ratio
p: probability value
SD: Standard deviation
SDM-Q-9: 9-item Shared Decision Making Questionnaire
t: t statistic
